# *In Vitro* Cytotoxicity of a New Nano Root Canal Sealer on Human Gingival Fibroblasts

**DOI:** 10.22037/iej.2017.43

**Published:** 2017

**Authors:** Maryam Javidi, Parisa Dastmalchi, Mina Zarei, Maryam Shayani Rad, Ahmad Ghorbani

**Affiliations:** a *Dental Materials Research Centre, Mashhad University of Medical Sciences, Mashhad, Iran; *; b *Student Research Committee, Mashhad University of Medical Sciences, Mashhad, Iran; *; c *Pharmacological Research Center of Medicinal Plants, Mashhad University of Medical Sciences, Mashhad, Iran*

**Keywords:** Cytotoxicity, Human Gingival Fibroblast, MTT assay, Nano, Sealer

## Abstract

**Introduction:**

The aim of this *in vitro* study was to evaluate the cytotoxicity of a new nano zinc-oxide eugenol (NZOE) sealer on human gingival fibroblasts (HGFs) compared with Pulpdent (micro-sized ZOE sealer) and AH-26 (resin-based sealer).

**Methods and Materials:**

The Pulpdent, AH-26, and NZOE sealers were prepared and exposed to cell culture media immediately after setting, and 24 h and one week after setting. Then, the primary cultured HGFs were incubated for 24 h with different dilutions (1:1 to 1:32) of each sealer extract. Cell viability was evaluated by methyl thiazolyl diphenyl tetrazolium bromide (MTT) assay. The results were compared using two-way analysis of variance followed by Tukey’s post hoc test. The level of significance was set at 0.05.

**Results:**

All sealer extracts, up to 32 times dilutions, showed cytotoxicity when exposed to HGF immediately after setting. The extracts obtained 24 h or one week after setting showed lower cytotoxicity than extracts obtained immediately after setting. At all setting times, NZOE showed lower cytotoxicity than Pulpdent and AH-26. While one-week extracts of NZOE had no significant effect on the viability of HGF at dilutions 1:4 to 1:32, both Pulpdent and AH-26 decreased the cell viability at dilutions of 1:4 and 1:8.

**Conclusion:**

NZOE exhibited lower cytotoxicity compared to Pulpdent and AH-26 on HGF and has the potential to be considered as a new root canal filling material.

## Introduction

The purpose of using sealer for obturation of the root canal system is to prevent penetration of microorganisms and their byproducts. However, sealer is in direct contact with periapical tissues and may cause inflammation, tissue degeneration and delay in wound healing. Therefore, the ideal root canal sealer should be non-cytotoxic, non-mutagenic and immunologically compatible with periapical tissues [1, 2]. Currently, a large variety of sealers with different formulas and physical properties are available for use. However, they all have their limitations. It is difficult to produce a sealer with proper physicochemical properties while being biocompatible for long-term. For many years, zinc oxide-eugenol (ZOE)-based sealers have been widely used in endodontic practice. These sealers have some limitations of their own. It has been shown that ZOE-based sealers release potentially cytotoxic concentrations of eugenol [2, 3]. Tai *et al.* [4] observed that ZOE-based root canal sealers are cytotoxic and genotoxic on Chinese hamster lung fibroblasts. Chandra *et al.* [5] showed that these sealers inhibit proliferation of kidney epithelial cells. Also, it has been shown that elutes prepared from ZOE sealers are cytotoxic for primary human periodontal ligament cells [6]. Further, there are some reports on possible neurotoxic effects of ZOE-based sealers [7, 8].

**Figure 1.**
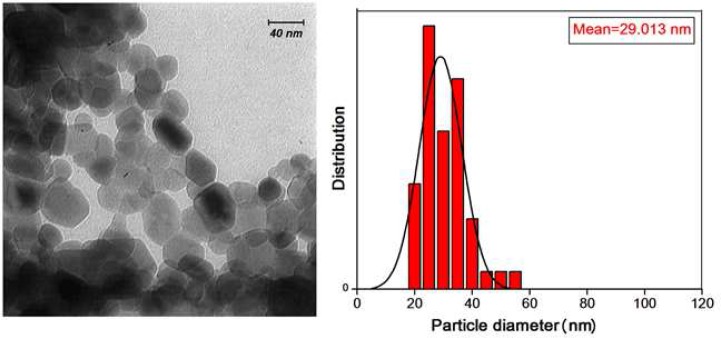
Transmission electron microscopy image (left) and corresponding particle size histogram (right) of nano powder in NZOE sealer

The use of nanotechnology has allowed many developments in dentistry and advances in oral-health-related nano material and therapeutic methods [9]. Nano technology is now used to produce a large number of dental materials, including light-cured restorative composite resins and their bonding systems, impression materials, ceramics, dental implant covering layers and fluoride mouthwashes. Some of the advantages of using nano particles in endodontic sealers include improving their physicochemical characteristics, enhancing the antibacterial property, decreasing microleakage, and increasing biocompatibility [10-12]. It has been shown that incorporating zinc oxide nano particles enhances the physicochemical characteristics (setting time, flow, solubility, dimensional stability and radiopacity) of Grossman sealer [12]. Kesler Shvero *et al.* [13] demonstrated that epoxy resin-based surfaces with cationic nano particles attracted and sacrificed *Enterococcus faecalis*. DaSilva *et al. *[14] showed that incorporating chitosan nano particles into ZOE sealer reduced the formation of biofilm within the sealer-dentin interface. Also, it has been reported that nano-ceramic sealer had better cytocompatibility than Endoseal MTA considering the effects on cell spreading and proliferation [15]. 

In previously published articles, we introduced a new nano-sized zinc oxide-eugenol (NZOE) sealer that had microleakage less than Pulpdent and AH-26 root canal sealers. It showed better antibacterial property in comparison with Pulpdent and AH-26 sealers [16]. In an animal study, it was observed that the histocompatibility properties of NZOE were comparable to the above mentioned commercial sealers [17]. Also, the cytotoxicity of NZOE on murine L929 cell line was comparable to that of Pulpdent and was lower than AH-26 sealer [18]. Before testing clinically, a newly synthesized sealer should be critically tested for possible toxicity on human cells. Therefore, the aim of this study was to evaluate the cytotoxicity of NZOE sealer on human gingival fibroblasts isolated from healthy subjects.

## Materials and Methods

***Materials***

The epoxy resin-based sealer (AH-26, Dentsply, De Trey, Konstanz, Germany) and ZOE-based sealer (Pulpdent, Watertown, MA, USA) were used in this study. Dimethyl sulfoxide, gelatin (type B from bovine skin), penicillin-streptomycin solution, type-II collagenase and the powder of 3-(4, 5-Dimethyl-2-thiazolyl)-2, 5-Diphenyl-2H-tetrazolium bromide (MTT) were purchased from Sigma (St Louis, MO, USA). Dulbecco's Modified Eagles Medium (DMEM) and fetal bovine serum (FBS) were obtained from GIBCO (Grand Island, NY, USA).

***Preparation of NZOE sealer***

**Figure 2:**
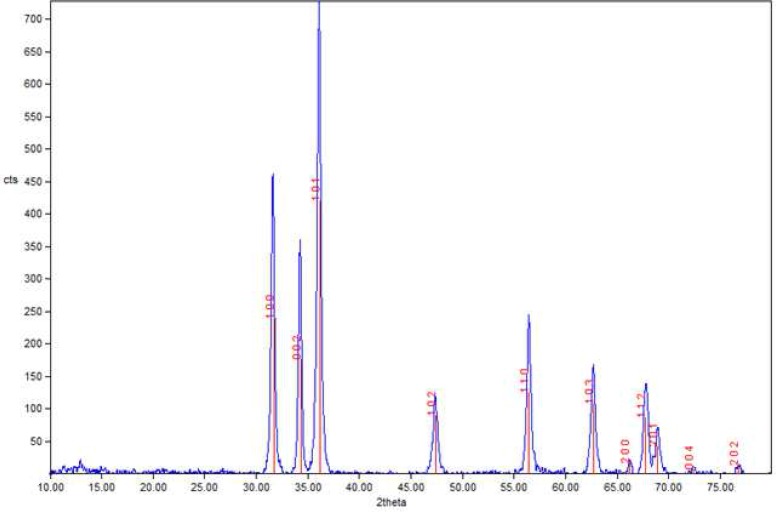
X-ray diffraction pattern of nano powder in the NZOE sealer

Nano sealer was prepared *via* a modified sol-gel method as described in previous work [16]. Briefly, a solution of gelatin was prepared by dissolving 10 g gelatin in 150 mL deionized water at 60^º^C. Then, an appropriate amount of zinc nitrate was dissolved in a minimum volume of deionized water at room temperature. The two prepared solutions were mixed and stirred for 8 h while the temperature was kept at 80 C. The prepared resin was calcined at 500^°^C, to obtain pure zinc oxide nano powders. The amount of nano-sized powder in nano powder composite was around 97%. Transmission electron microscopy (TEM) image and corresponding particle size histogram of nano powder are shown in Figure 1. The average size of the nano particles was about 30 nm. Crystallite size of nano powders which was calculated by applying FWHM peaks of X-ray diffraction pattern was about 18 nm (Figure 2).

***Isolation and culture of human fibroblasts***

Human gingival fibroblasts (HGFs) were obtained from healthy gingival tissue specimen of three volunteers who were undergoing oral surgery (third molar extraction) only for dentistry reasons in the Clinic of Dentistry, Mashhad University of Medical Sciences, Mashhad, Iran. All procedures performed in this study were in accordance with the ethical standards of Mashhad University of Medical Sciences and with the 1964 Helsinki declaration and its later amendments or comparable ethical standards. Informed consent was obtained from each volunteer. The tissue specimen was transferred to the laboratory in sterile phosphate buffered saline (PBS) containing 100 units/mL penicillin and 100 µg/mL streptomycin. After washing with sterile PBS, the tissue was cut into small pieces and digested in PBS containing collagenase (two mg/mL) under shaking (60 cycles/min) at 37^°^C [19, 20]. After centrifugation, the pelleted cells were suspended in DMEM medium supplemented with 10% FBS and antibiotics and then seeded into tissue culture flask. After 48 h, the non-adherent cells were discarded by changing the medium and the anchorage-dependent cells were preserved. Subconfluent cells were harvested and expanded further through three passages. 

***Preparation of sealer extract***

Three types of sealer extract were obtained for each NZOE, Pulpdent, and AH-26 sealers: 1) extract obtained immediately after sealer setting, 2) extract obtained 24 h after setting and 3) extract obtained one week after setting. First, all the NZOE, Pulpdent, and AH-26 sealers were prepared according to their manufacturers’ instructions and the samples of each one were separately placed into 24-well cell culture plate. Two wells were considered for each sealer and the volume of each sealer in each well was 16 mm in diameter and 2 mm in high. Three plates were prepared in this manner. In the case of the first plate, immediately after setting each well was covered with 2.5 mL DMEM supplemented with antibiotics and incubated in the dark for 24 h at 37^°^C [21]. For the second and third plates, the medium was added to the wells after 24 h and one week of sealer setting, respectively, and then incubated for 24 h at 37^°^C. After incubation, the conditioned media (sealer extracts) were collected. These original extracts (1:1 dilution) were passed through 0.22 µm filters and then serially diluted in fresh DMEM supplemented with antibiotics and 10% FBS. Different dilutions (1:1, 1:2, 1:4, 1:8, 1:16 and 1:32) of each sealer were used for cytotoxicity assay [18].

***Cell culture and treatment***

The HGFs at the subconfluent stage were harvested from culture flask and their viability was checked by trypan blue exclusion test. Then, the cells were seeded in 96-well culture plate containing DMEM medium supplemented with 10% FBS, 100 U/mL penicillin, and 100 µg/ mL streptomycin. After 24 h, the culture medium was replaced by fresh one containing varying dilutions (1:1 to 1:32) of extracts from AH-26, Pulpdent, or NZOE sealers. Untreated fibroblasts that were incubated in medium containing no sealer extract were considered as control cells. Then, the treated and control cells were further incubated at 37˚C in an atmosphere containing 5% CO_2_ for 24 h. 

***MTT cell viability assay***

The MTT assay is based on the reduction of the tetrazolium salt into formazan crystals by mitochondrial enzymes of living cells. After 24 h incubation of the cells with sealers, the MTT solution (5 mg/mL PBS) was added to each well of culture plate to make a final concentration of 0.5 mg/mL. After 2 h, the supernatant of each well was removed and the resulting formazan was dissolved in 200 μL dimethyl sulfoxide. The optical density of formazan dye was read at 545 nm against 630 nm as background by Elisa reader (Awareness Technology Inc., Palm City, FL, USA). The percentage of cell viability in each well was calculated relative to control cells set to 100% [22, 23]. Each assay was performed in triplicate and repeated independently two times.

***Statistical analysis***

The results were compared using the two-way analysis of variance (ANOVA) followed by Tukey’s post hoc test. *P*-values less than 0.05 were considered statistically significant.

## Results

***Cytotoxicity of sealers immediately after setting***

**Figure 3:**
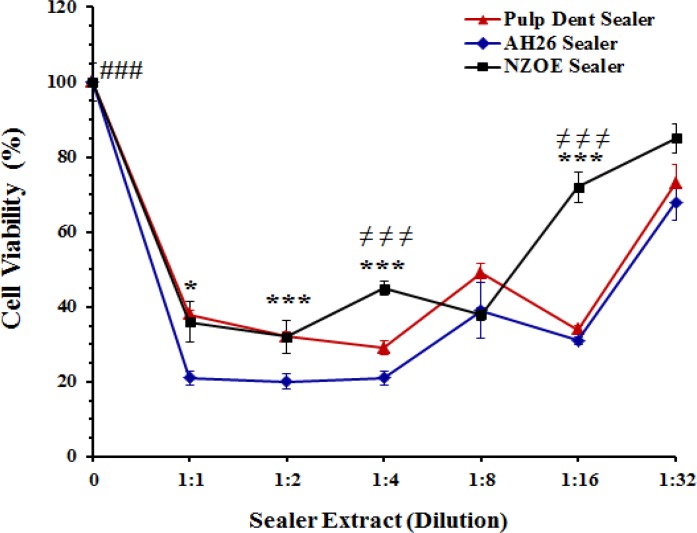
Effects of extracts obtained immediately after setting of Pulpdent, AH-26, and NZOE sealers on the viability of human gingival fibroblasts.

As shown in Figure 3, all samples in this group had cytotoxic property up to 32 times dilutions. Viability of cells treated with 1:1, 1:2, 1:4, 1:8, 1:16, and 1:32 dilutions of AH-26 extract was significantly decreased from 100±5% (control) to 21±2%, 20±2%, 21±2%, 39±7.5%, 31±1%, and 68±5%, respectively (*P*<0.001). The percent of cell viability at presence of 1:1, 1:2, 1:4, 1:8, 1:16, and 1:32 dilutions of Pulpdent extract was 38±1%, 32±1.3%, 29±1.8%, 49±2.6%, 34±1%, and 73±5%, respectively (*P*<0.001 versus control). Statistical analysis showed that the cytotoxicity of NZOE was lower than that of AH-26 (*P*<0.001) and Pulpdent (*P*<0.01) sealers. At dilutions of 1:4 (45±2%), 1:16 (72± %) and 1:32 (85±4%), viability of cells treated with NZOE was significantly (*P*<0.001) higher than cells treated with AH-26 or Pulpdent sealers.

**Figure 4:**
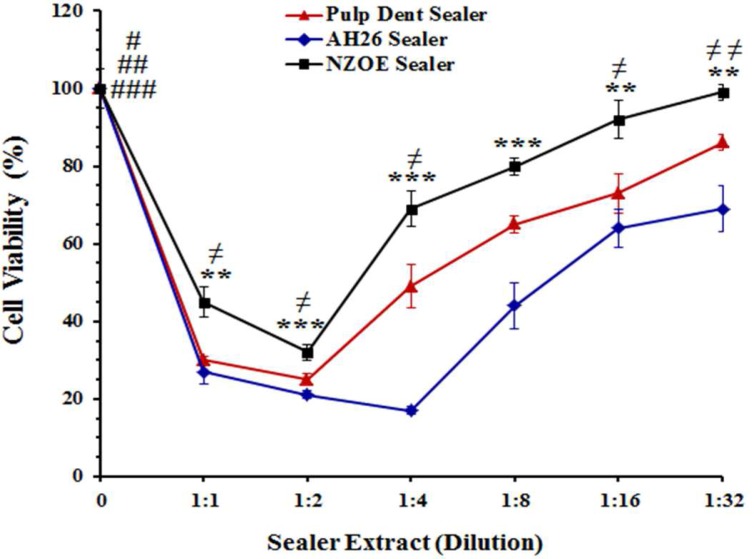
Effects of extracts obtained 24 h after setting of Pulpdent, AH-26, and NZOE sealers on viability of human gingival fibroblasts

**Figure 5:**
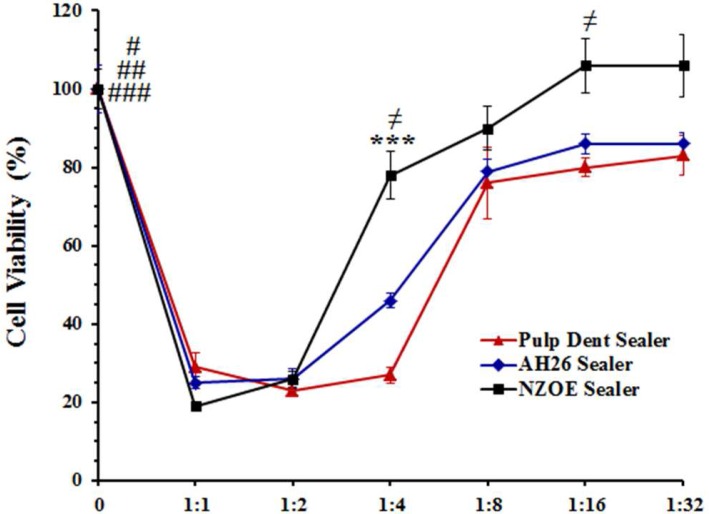
Effects of extracts obtained 1 week after setting of Pulpdent, AH-26, and NZOE sealers on viability of human gingival fibroblasts

***Cytotoxicity of sealers 24 h after setting***

After 24 h of setting, non-diluted extracts obtained from Pulpdent, AH-26 and NZOE sealers significantly decreased viability of fibroblasts from 100±5% (control) to 30±1% (*P*<0.001), 27±3% (*P*<0.001), and 45±4% (*P*<0.001), respectively (Figure 4). The cytotoxicity effect of extracts decreased at dilutions of 1:4 to 1:32. Regarding NZOE extract, the level of cell viability at dilutions of 1:8, 1:16, and 1:32 was 80±2.2%, 92±5%, and 99±2%, respectively, which was not statistically different with that of control cells. At all dilutions, the lowest and the highest viability were seen in cells treated with AH-26 and NZOE, respectively. The cytotoxicity of NZOE was lower than that of AH-26 (*P*<0.001) and Pulpdent (*P*<0.001) sealers.

***Cytotoxicity of sealers one week after setting***

Again, the cytotoxicity of NZOE sealer was lower than that of AH-26 (*P*<0.001) and Pulpdent (*P*<0.001) sealers (Figure 5). While NZOE at dilutions of 1:4 to 1:32 had no significant effect on the viability of fibroblasts, both Pulpdent and AH-26 significantly decreased the cell viability at dilutions of 1:4 (*P*<0.001) and 1:8 (*P*<0.05). Also, the cytotoxicity of NZOE was lower than Pulpdent or AH-26 sealers at dilutions of 1:4 to 1:32. The level of cell viability at dilutions of 1:4, 1:8, 1:16, and 1:32 was 78±6%, 90±5.5%, 90±5.5%, and 106±7%, respectively. 

## Discussion

Root canal filling materials are used to minimize the risk of infection and to promote the healing of periapical tissues [24]. Since these materials can come in contact with surrounding tissues, they should be nontoxic and non-carcinogenic. Contact with tissues may occur because of overfilling, extrusion or leak of diffusible substances into periradicular tissues. When this happened, the affected area undergoes inflammation and destruction which may lead to tenderness and pain [25]. Therefore, when a new root canal sealer is introduced, it should be critically evaluated for possible cytotoxicity. According to our data, all the extracts of Pulpdent, AH-26, and NZOE obtained immediately after setting had a cytotoxic effect up to 32 times dilutions. This finding again supports previous reports on cytotoxicity of different classes of root canal sealers [1, 6, 24-26].

Clinically, the sealers are placed into the root canal in a freshly mixed stage and therefore it is possible that potentially toxic constituents leak into tissue fluids. In the present study, an attempt was made to simulate a condition with the maximum cytotoxic effect of the sealers in the human body. Therefore, the sealers were exposed to the culture media immediately after setting and incubated for 24 h to ensure that all the toxic agents would be released into the extract. In comparison, among all three sealers, AH-26 had the highest and NZOE had the lowest cytotoxicity. Similarly, in our previous study on murine L929 fibroblasts, we observed that the cytotoxicity of NZOE was lower than AH-26 sealer [18]. In that study, we tested the toxicity of NZOE sealer only immediately after setting. In the present work, we tested also the toxicity of sealers 24 h and one week after setting. Regarding sealer extracts obtained 24 h after setting, again AH-26 had the highest and NZOE had the lowest cytotoxicity on gingival-derived cells. Also, while extract of NZOE obtained one week after setting had no significant effect on the viability of fibroblast at dilutions 1:4 to 1:32, both Pulpdent and AH-26 sealers decreased the cell viability at dilutions of 1:4 and 1:8. Again, the cytotoxicity of NZOE was lower than Pulpdent and AH-26 sealers at dilutions of 1:4 to 1:32. The present data are consistent with those of Bae *et al.* [27] who showed that a ZOE-based sealer (EWT) at dilutions of 1:2 to 1:16 is less toxic than that of AH-26 sealer. In addition, Huang *et al.* [7] reported that the cytotoxicity of AH-26 sealer at 24 h, 7 days, and 14 days after setting is greater than that of Canals (a ZOE -based sealer). Although our results regarding extracts obtained 24 h after setting are in agreement with those of Huang *et al. *[7], we didn’t find any significant differences between the level of toxicity of AH-26 and Pulpdent. This discrepancy may come from the difference in the methods of cell treatment. In that study the materials have been placed in direct contact with cells; however, in the present work, the cells were incubated with the extract of sealers. Since direct placement of the sealer in the culture dish may cause physical injuries to cells and increases the risk of bacterial contamination, in the present study like several other studies, sealer extract technique was used [6, 7, 24].

It is well known that the composition of endodontic sealers has an important influence on their biocompatibility. In the present study, we observed that NZOE has less cytotoxicity compared to Pulpdent sealer suggesting that incorporating zinc oxide nanoparticles decreases the cytotoxicity of ZOE-based sealers. In line with our findings, a recent report by Collado‐González *et al.* [15] demonstrated that the nano ceramic sealer had better cytocompatibility than other calcium silicate-based endodontic sealer, Endoseal MTA. As far as we know, our study was the first to evaluate the cytotoxicity of a NZOE sealer and further studies on this subject are needed.

The advantage of using permanent cell lines in toxicity assay of dental materials include no ethical issues, controlled experimental situations, low costs and rapid performance [28]. However, the main limitation of toxicity assay in cell culture condition is the lack of simulation of the *in vivo* situation. In addition, cell lines of various origins may show different responses in the presence of endodontic sealers. In the present work, we used primary cultured (not cell line) fibroblasts isolated from gingival tissue specimen of healthy volunteers. Gingival fibroblasts are among the best-used cells for *in vitro* cytotoxicity assays of root canal filling materials. They have common connective tissue origin with periodontal membrane fibroblasts, and therefore their results are closer to clinical situation [25, 29].

## Conclusion

The results of present study showed that the synthesized nano sealer exhibits lower cytotoxicity on gingival fibroblasts compared to Pulpdent and AH-26 sealers. Therefore, it has the potential to be considered as a new root canal filling material in endodontics. Further studies are suggested before NZOE sealer can be recommended for clinical application.
